# Complication rate and outcomes of laryngeal cuneiformectomy in dogs with advanced laryngeal collapse

**DOI:** 10.1111/vsu.14270

**Published:** 2025-06-02

**Authors:** Alexander J. Chan, Nai‐Chieh Liu, Jane F. Ladlow

**Affiliations:** ^1^ Bristol Veterinary Specialists Bristol UK; ^2^ Institute of Veterinary Clinical Science, School of Veterinary Medicine National Taiwan University Taipei Taiwan; ^3^ Granta Veterinary Specialists Cambridge UK; ^4^ Department of Veterinary Medicine University of Cambridge Cambridge UK

## Abstract

**Objective:**

To describe the complication rate and outcomes of dogs undergoing multilevel airway surgery for brachycephalic airway syndrome (BOAS) with and without the addition of uni‐ or bilateral cuneiformectomy.

**Study design:**

Retrospective study.

**Animals:**

A total of 180 dogs undergoing BOAS surgery: 94 dogs undergoing modified multilevel surgery (non‐PC); 86 additionally undergoing cuneiformectomy (PC).

**Methods:**

Case records from the University of Cambridge and Animal Health Trust databases between 2014 and 2021 were analyzed including data on laryngeal collapse grade, respiratory functional grading scores, BOAS index, hospitalization length and complications.

**Results:**

Neither the incidence risk of overall (non‐PC = 19.4%, PC = 16.3%, *p* = .758), nor major (non‐PC = 7.4%, PC = 11.6%, *p* = .482) complications differed between non‐PC and PC dogs. Median hospitalization duration (non‐PC = 1 day, PC = 1 day) did not differ between the two groups (*p* = .743). Both BOAS grade (median reduction = 1, *p* < .0001) and BOAS index (median reduction = 28.5%, *p* < .0001) reduced in dogs that underwent cuneiformectomy. Lower BCS was associated with increased postoperative complications (odds ratio = 0.452, *p* = .004) when preoperative BOAS grade and gender were controlled.

**Conclusion:**

Cuneiformectomy was not associated with a higher incidence risk of complications than multilevel BOAS surgery alone. Significant improvements in respiratory parameters were observed following cuneiformectomy in addition to multilevel airway surgery.

**Clinical significance:**

Cuneiformectomy represents a safe and effective adjunctive technique to manage higher grade laryngeal collapse in dogs with BOAS.

**Abbreviations**
BCS: body condition score
BOAS: brachycephalic obstructive airway syndrome
CI: confidence interval
CMS: convential multilevel surgery
LATE: laser assisted turbinectomy
MMS: modified multilevel surgery
non‐PC: non partial cuneiformectomy
OR: odds ratio
PC: partial cuneiformectomy
WBBP: whole body barometric plethysmography

## 1 INTRODUCTION

Brachycephalic obstructive airway syndrome (BOAS) is a disorder affecting breeds with short and wide skulls and muzzles where the soft tissues of the upper respiratory tract are compressed into a proportionally smaller volume of space.^1^, ^2^ Typical abnormalities are breed specific and include stenotic nares, oversized soft palates, reduced nasopharynx, aberrant nasal turbinates, hypoplastic trachea, tonsillar hypertrophy, macroglossia, laryngeal ventricle eversion and in more severe cases, laryngeal collapse. These changes obstruct respiration leading to clinical signs such as stertor or stridor, dyspnea, cyanosis, regurgitation or vomiting,^3^ heat and exercise intolerance and collapse. Clinical signs can progress over time and are exacerbated by obesity owing to an increase in the amount of tissue surrounding the airways and particularly the tongue.^4^, ^5^

Laryngeal collapse occurs as the cartilages lose their rigidity as a result of chronically elevated intraluminal airway pressures^6^ and is associated with audible stridor on clinical examination.^7^ It is staged as follows^6^, ^8^, ^9^: (stage I) laryngeal ventricle eversion, (stage II) loss of arytenoid cartilage rigidity with medial displacement of the cuneiform processes, (stage III) corniculate process collapse. Higher grades (II or III) are reported in 50%–63% of BOAS cases.^10^, ^11^, ^12^ For dogs that have developed laryngeal collapse, there is debate in the literature with regards to the effect on prognosis. For dogs with lower grade laryngeal collapse, surgical treatment via ventriculectomy can result in considerable improvement in clinical signs.^13^ Nevertheless, there is some evidence to suggest that dogs undergoing ventriculectomy are more likely to develop postoperative complications than those not undergoing laryngeal surgery.^14^ However, many studies consider higher stages of collapse to confer a negative prognosis.^10^, ^15^, ^16^, ^17^ Primary developmental laryngeal pathology such as chondromalacia may also contribute to the progression of the disease, particularly in the pug.^18^

The severity of BOAS may be quantified by validated methods such as functional grading^19^ or whole‐body barometric plethysmography (WBBP).^19^, ^20^ Both techniques are non‐invasive and attempt to provide an objective measurement of disease severity to circumvent the issue of variability in clinical assessment. For functional grading, dogs are graded from 0 (unaffected), to 3 (severely affected). Grades 0 and 1 are regarded as having clinically insignificant signs, whereas grades 2 and 3 dogs are significantly affected by BOAS.^20^ From WBBP readings, a BOAS index can be obtained, producing values from 0%–100%, with higher scores indicating more severe disease. Dogs with higher grade laryngeal collapse have been found to have higher mean BOAS indices (84%) than those with just ventricle eversion (70%).^17^

**FIGURE 1:**
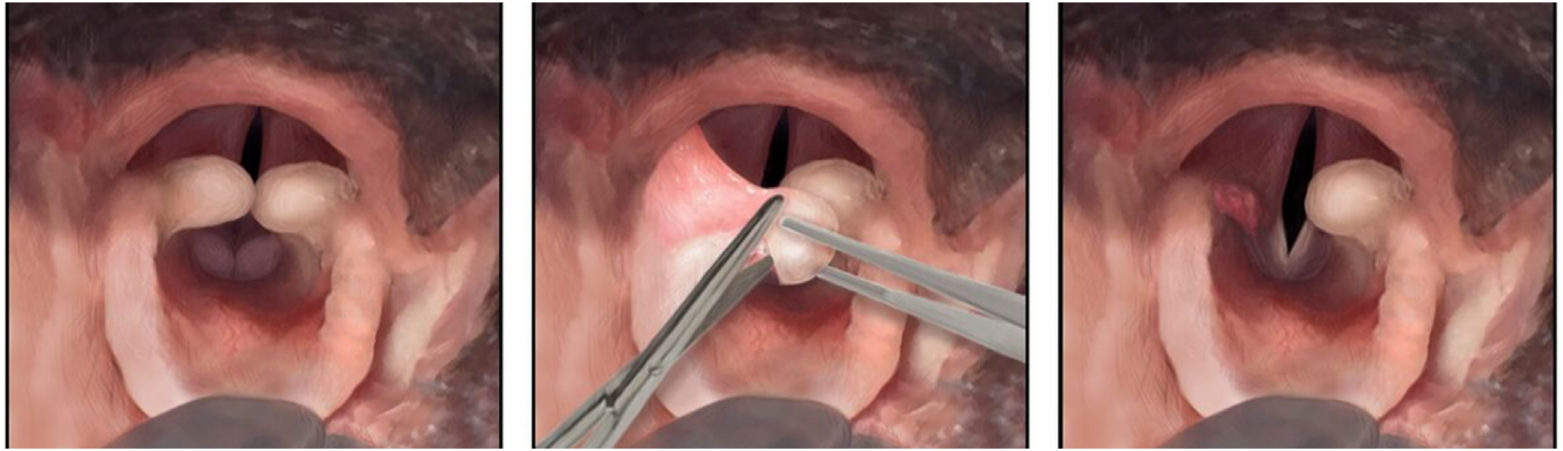
Partial cuneiformectomy: Surgical technique demonstrating right‐sided partial cuneiformectomy. Image courtesy of Nai‐Chieh Liu.

**FIGURE 2:**
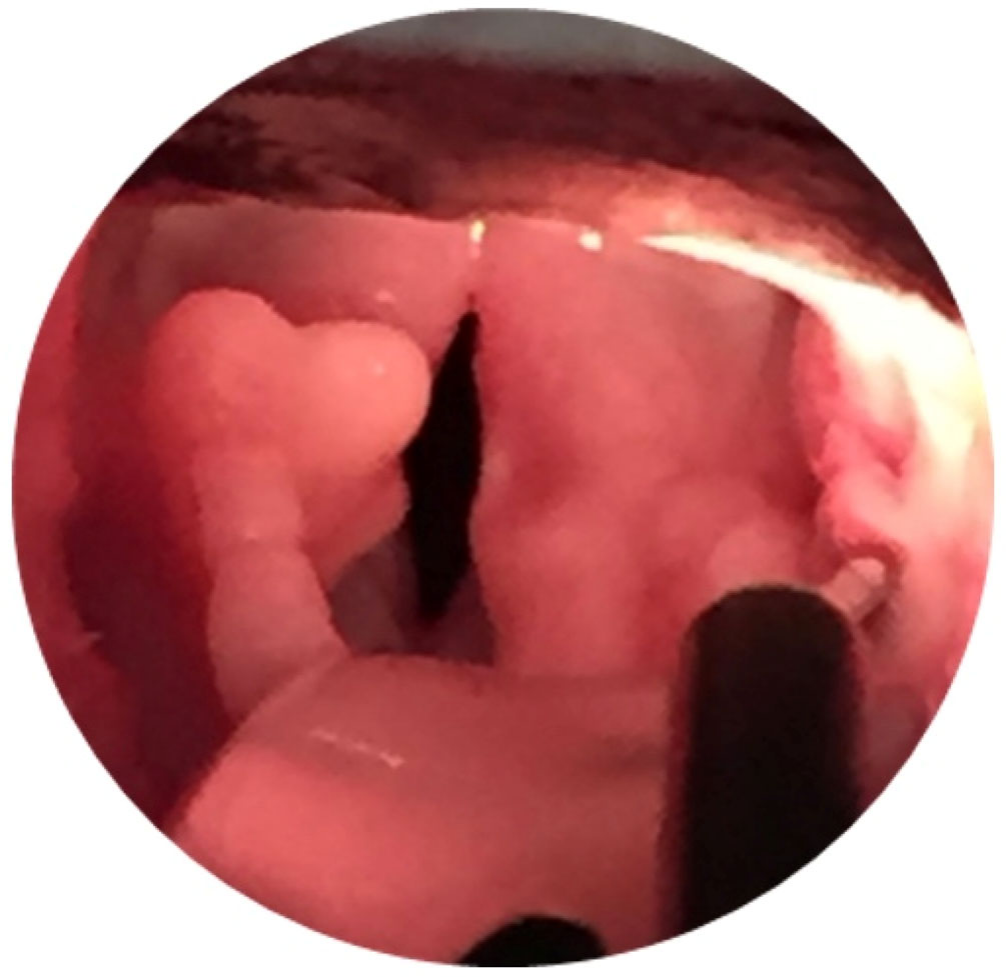
Postoperative photo of left‐sided partial cuneiformectomy in a Pug. Image courtesy of Jane Ladlow.

Many features of BOAS are amenable to surgery and though there are multiple techniques described, conventional multilevel surgery (CMS) typically includes alaplasty, staphylectomy, partial tonsillectomy and laryngeal ventriculectomy if the latter is indicated. More recently, a modified approach (MMS) consisting of alavestibuloplasty, folding flap palatoplasty, partial tonsillectomy, ventriculectomy and partial cuneiformectomy (PC) (in dogs with grade 2 or 3 laryngeal collapse; Figures 1 and 2) was described by Liu and colleagues,^17^ using techniques derived from Oechtering. One study reported improvements in median BOAS index from 75.8% to 63.2% after airway surgery with dogs either undergoing CMS or MMS.^17^ Surgical intervention has the potential to significantly improve the quality of life of many dogs affected by BOAS. Nevertheless, respiratory function, as measured by BOAS indices, remains compromised in approximately 60% of dogs postoperatively to some degree.^17^ For these refractory cases laser assisted turbinectomy (LATE) may be of benefit.^17^, ^21^

Though traditionally permanent tracheostomy^22^ has been used to manage cases of advanced laryngeal collapse in BOAS dogs, the condition may be treated directly via cricoarytenoid lateralization with thyroarytenoid caudolateralisation^23^, ^24^ or alternatively via partial cuneiformectomy^6^, ^25^ as previously discussed. The latter negates the requirement for repositioning after the other intra‐oral components of BOAS multilevel surgery, such as palatoplasty or tonsillectomy, have been performed. Partial laryngectomy reduces dynamic obstruction and widens the rima glottidis by approximately 70%–80%.^26^ Healing occurs by granulation and epithelialization with the process completed by 14 days.^27^ More recently, arytenoidectomy has been performed using laser photoablation.^28^ Both ventriculectomy and laryngoplasty have been reported to be associated with greater perioperative risk^8^ and the benefit of performing partial laryngectomy has been questioned with one study finding that 50% of dogs with laryngeal collapse undergoing laryngectomy had airway disease as their primary cause of death.^29^ It was suggested that poor results may have been attributable to aspiration following unilateral laryngectomy and due to an obstructive laryngeal webbing forming from granulation tissue at the dorsal and ventral commissures if bilateral laryngeal surgery was performed. Notably, this study involved the excision of sections of the vocal folds and the cuneiform and corniculate cartilage whereas the MMS technique is limited to resection of the cuneiform process alone. Another study^17^ found that among dogs with advanced laryngeal collapse the median postoperative BOAS indices did not differ significantly between dogs that underwent laryngeal surgery versus those that did not. However, this may be attributable to type II error due to the small number of dogs undergoing cuneiformectomy in this study. Additionally, surgery in advanced cases of collapse may necessitate temporary tracheostomy either prophylactically at the time of surgery, or in cases of postoperative respiratory distress in dogs with significant airway inflammation and edema.^12^

The purpose of this study was primarily to assess the incidence risk of complications of cuneiformectomy in treating laryngeal collapse, but also to examine effect on functional grades and BOAS indices when performed in addition to MMS. The null hypothesis was that there would be no difference in the incidence risk of complications between dogs undergoing MMS without PC and the group undergoing MMS with PC.

## 2 MATERIALS AND METHODS

### 2.1 Study design

Case records from client owned brachycephalic dogs that had undergone MMS with without cuneiformectomy between 2014 and 2021 were sourced from the University of Cambridge and Animal Health Trust BOAS databases including data concerning age, gender, neutered status, breed, body condition score (BCS: 1–9 scale), laryngeal collapse status, pre‐ and postoperative BOAS functional grades and BOAS indices from plethysmography, surgical procedures, major and general complications and hospitalization duration postoperatively. The procedures required to perform respiratory functional grading and plethysmography have been extensively described in previous studies.^19^, ^20^ The inclusion criteria for the study were brachycephalic dogs that had undergone surgical treatment of BOAS. Dogs undergoing cuneiformectomy were named the “PC group”; dogs that did not undergo cuneiformectomy were assigned to the “non‐PC group”. Dogs were excluded if they presented with non‐BOAS related airway disease at pre‐ or postoperative examination. For dogs that underwent bilateral staged cuneiformectomy, only measurements prior to and following the first procedure were included. Recording of BOAS functional grades and indices were carried out at the preoperative assessment and at postoperative rechecks performed between six and 12 weeks postoperatively. All dogs underwent tonsillectomy, modified folding flap staphylectomy and alavestibuloplasty. The decision to perform cuneiformectomy was case dependent according to the perceived benefit of performing the procedure. The collapsed cuneiform process was grasped with atraumatic forceps and the medial aspect resected with microvascular scissors (Figures 1 and 2). In English and French Bulldogs, a branch of the laryngeal artery is commonly encountered ventrolaterally. Should this occur, hemostasis is performed with bipolar electrocautery. Following cuneiformectomy, the excision site was packed with an adrenaline‐soaked cotton bud for the remainder of the airway procedure. The criteria for major complications were those requiring surgical intervention or resulting in severe dyspnea, postoperative death or euthanasia. Severe dyspnea was defined as requiring intubation to maintain oxygen saturation. Minor complications were any complications that did not meet these criteria, for example worsening regurgitation or mild hemorrhage from the surgical sites not requiring surgical intervention. Complications were counted once per dog; consequently, for example a dog undergoing severe dyspnea leading to euthanasia was only classified as having one single, major complication. Ethical consent (CR 63) was acquired for the data to be used for all dogs in the study by the Department of Veterinary Medicine, University of Cambridge. Follow up was carried out until six to 12 weeks postoperatively or until euthanasia. This consisted of physical examination with functional grading and plethysmography where possible, in addition to medical records and communications with owners and referring clinicians.

### 2.2 Statistical analysis

All statistical analyses were performed using the statistical package “R” (version 3.5.3).^30^ The significance level was set at *p*‐value < .05. The normality of continuous variables was tested using the Kolmogorov–Smirnov test and frequency histogram. Independent sample *t*‐test or Mann–Whitney U tests were used to compare continuous variables (age, BOAS index, hospitalization duration) between the two groups, while categorical variables were compared using the chi‐squared or Fisher's exact test. For ordinal variables, the Mann–Whitney U test was used. To assess the associations between unilateral, staged or one stage bilateral cuneiformectomy and postoperative complications, chi‐squared tests were performed. Risk factor assessment for postoperative complications was conducted using logistic regression. A binary outcome for complications was defined and coded as follows: 0 = no new complications; 1 = had new complications postoperatively. The explanatory variables were breed, age, gender, BCS, stage of laryngeal collapse, preoperative BOAS grade, whether cuneiformectomy was performed and hospitalization length. BCS data was handed as semicontinuous data. Variables with a Wald test *p*‐value < .25 on univariate logistic regression were selected for the multivariate analysis by using the method of purposeful selection. Backward stepwise model‐selection based on Akaike's information criteria was used to obtain the best‐fit model. To compare the pre‐ and post‐surgery BOAS grade and BOAS index, Wilcoxon signed‐rank tests were used. Multicollinearity among independent variables was evaluated using variance inflation factors, confirming that no predictors exhibited high collinearity. The stage of laryngeal collapse was removed from the analysis due to its high collinearity with cuneiformectomy. The linearity assumption between continuous predictors and the log‐odds was assessed by using partial residual plots, ensuring that the assumption was met. Outliers and influential points were identified and evaluated using Cook's distance and leverage statistics. Finally, the goodness‐of‐fit was verified using the Hosmer‐Lemeshow test to confirm that the model adequately fit the data.

## 3 RESULTS

A total of 180 dogs were included comprising 86 undergoing cuneiformectomy and 94 that did not. Baseline characteristics of the two groups are shown in Table 1. Seven breeds were represented; Pugs (*n* = 62), French Bulldog (*n* = 99), English Bulldog (*n* = 11), Cavalier King Charles spaniel (*n* = 2), Boston Terrier (*n* = 2), Norwich Terrier (*n* = 1), Pekingese (*n* = 1) and two cross breeds (Pug × Cavalier King Charles spaniel; Pug × French Bulldog). Ages ranged between 4 months and 10.2 years (median 25 months). There were 76 females (36 entire, 40 neutered) and 104 males (62 entire, 42 neutered) participating in the study. Pug breed (*p* < .0001), older age (*p* = .002) and female dogs (*p* = .002) were over‐represented in the PC group. In both groups the median hospitalization duration (non‐PC = 1 day, PC = 1 day, range, 1–8 days) did not differ between groups. Cuneiformectomy was not associated with an increased duration of hospitalization (*p* = .743). Laryngeal collapse data was available for all 180 dogs included in the study. Laryngeal collapse was present in 57/94 of the non‐PC group (93% were grade I and 7% grade II). For the PC group, 79% were grade II and 21% grade III (Table 1). As such, the grade of laryngeal collapse was higher in the PC group (*p* < .0001).

**TABLE 1:** Patient characteristics and results of group comparisons in dogs who underwent MMS for BOAS with and without PC.

	MMS without PC (*n* = 94)	MMS with PC (*n* = 86)	*p*‐value
Breeds	Boston Terrier (1); English Bulldog (8); cross‐breed – French bulldog × Pug (1); French bulldog (72); Pug (12)	Boston Terrier (1); English Bulldog (3); CKCS (2); cross‐breed – Pug × CKCS (1); French Bulldog (27); Norwich Terrier (1); Pekingese (1); Pug (50)	<.0001
Median age (months [range])	23.5 [4–122]	30 [6–114]	.002
Gender (female, %)	29/94 (30.9%)	47/86 (54.7%)	.002
Stage of laryngeal collapse	Stage 0: 37/94 (39.4%) Stage I: 53/94 (56.4%) Stage II: 4/94 (4.3%) Stage III:0/94 (0%)	Stage 0: 0/86 (0%) Stage I: 0/86 (0%) Stage II: 68/86 (79.1%) Stage III:18/86 (20.9%)	<.0001
Preoperative BOAS Functional grade (*N* = 166)	Grade I: 7/91 (7.7%) Grade II: 66/91 (72.5%) Grade III: 18/91 (19.8%)	Grade I: 1/75 (1%) Grade II: 30/75 (40%) Grade III: 44/75 (59%)	<.0001
Postoperative BOAS Functional grade (*N* = 118)	Grade 0: 9/67 (13.4%) Grade I: 37/67 (55.2%) Grade II: 21/67 (31.3%) Grade III:0/67 (0%)	Grade 0: 4/51 (7.8%) Grade I: 26/51 (51%) Grade II: 18/51 (35.1%) Grade III:3/51 (5.9%)	.144
Median preoperative BOAS index (% [range]) (*N* = 99)	68 [34–100]	79.7 [14.7–87]	.004
Median postoperative BOAS index (% [range]) (*N* = 95)	45.9 [58–100]	60.2 [33–97.8]	.041
Hospitalization length (days [range])	1 [1–8]	1[1–8]	.743
Overall complication rate (%)	19.1	16.3	.758
Major complication rate (%)	7.4	11.6	.482

Abbreviations: BOAS, brachycephalic obstructive airway syndrome; CKCS, Cavalier King Charles spaniel; MMS, modified multilevel surgery; PCS, partial cuneiformectomy.

### 3.1 Incidence of complications

The overall incidence risk of complications (non‐PC = 19.4%, PC = 16.3%, X‐squared = 0.094, df = 1, *p* = .758) and the incidence risk of major complications (non‐PC = 7.4%, PC = 11.6%, X‐squared = 0.494, df = 1, *p* = .482) did not differ between dogs with and without cuneiformectomy. The most common complication was regurgitation, with worsening signs or regurgitation that was not previously reported, occurring in 8/94 (8.5%) non‐PC and 5/86 (5.8%) PC groups. Of these dogs, nine were French Bulldogs, two were English Bulldogs and two were Pugs. The incidence risk for both overall and general complications were highest in underweight dogs and lowest in overweight dogs (Table 2). The incidence risk of mortality prior to discharge were not different between the two groups (1/94 [1%] in the non‐PC group and 3/86 [3.5%] in the PC group, *p* = .35). One dog in the non‐PC group was euthanized following dehiscence of the palatoplasty site with regurgitation and hemorrhage. Of the three dogs undergoing cuneiformectomy that failed to survive to discharge, one was euthanized due to postoperative neurological signs unrelated to laryngeal surgery; the remaining two cases developed aspiration pneumonia following dyspnea and regurgitation (Table 3).

**TABLE 2:** The association between body condition score and incidence risk of complications.

	Body condition score (1–9)		
	<4	4–5	>5
Number of dogs undergoing MMS without PC	5	58	24
Number of dogs undergoing MMS with PC	4	29	40
Number of overall complications	4	18	4
Incidence risk of overall complications (%)	44.4	20.7	6.3
Number of major complications	3	10	2
Incidence risk of major complications (%)	33.3	11.5	3.2

Abbreviations: MMS, modified multilevel surgery; PC, partial cuneiformectomy.

**TABLE 3:** Major complications.

Case	Gender and neutered status	Age/months	Breed	Group	Major complication	Outcome
39	MN	12	French Bulldog	MMS without PC	Dyspnea; temporary tracheostomy performed postoperatively	Discharged
30	MN	4	French Bulldog	MMS without PC	Perforation of nasal mucosa when performing staphylectomy; regurgitation and hemorrhage 2 days postoperatively; cyanosis; dehiscence of staphylectomy site. Euthanasia performed	Euthanized
42	FE	7	Bulldog	MMS without PC	Dyspnea	Discharged
75	FE	20	French Bulldog	MMS without PC	Dyspnea	Discharged
90	FN	15	Pug	MMS without PC	Dyspnea	Discharged
107	ME	12	French Bulldog	MMS without PC	Regurgitation, worsening anorexia and dyspnea	Discharged
152	FE	48	French Bulldog	MMS without PC	Inflammatory laryngitis and stridor developed postoperatively. Temporary tracheostomy and revision surgery performed.	Discharged
175	ME	76	French Bulldog	MMS without PC	Dyspnea	Discharged
7	FE	26	Pug	MMS with PC	Hemorrhage from surgical wounds and dyspnea	Discharged
18	ME	37	Bulldog	MMS with PC	Regurgitation, hemorrhage, dyspnea and aspiration pneumonia	Discharged
22	ME	48	French Bulldog	MMS with PC	Cardiac arrest on recovery; subsequent airway obstruction due to suspected laryngospasm, tracheostomy tube placed. Seizures and elected for euthanasia. Post‐mortem diagnosis of ventriculomegaly and hydrocephalus	Euthanized
35	FN	15	Pug	MMS with PC	Dyspnea, temporary tracheostomy tube placed	Discharged
54	ME	24	Pug	MMS with PC	Dyspnea; tracheostomy tube placed	Discharged
60	FE	12	Pug	MMS with PC	Dyspnea, tracheostomy tube placed, aspiration pneumonia and euthanasia performed	Euthanized
80	FN	17	French Bulldog	MMS with PC	Dyspnea; tracheostomy tube placed, worsening regurgitation	Discharged
94	MN	28	French Bulldog	MMS with PC	Regurgitation, suspected aspiration pneumonia, Cardiopulmonary arrest three days postoperatively	Died
122	FN	37	Pug	MMS with PC	Dyspnea, temporary tracheostomy tube placed	Discharged
155	ME	30	French Bulldog	MMS with PC	Dyspnea, temporary tracheostomy tube placed	Discharged

Abbreviations: FE, female entire; FN, female neutered; ME, male entire; MMS, modified multilevel surgery; MN, male neutered; PC, partial cuneiformectomy.

Multivariate logistic regression analysis was performed on the independent variables of cuneiformectomy, BOAS grade, gender, body condition score (BCS) and age on the probability of developing major complications (Table 4). The best fit model included only preoperative BOAS grade, gender and BCS, with cuneiformectomy being excluded. Low BCS was associated with increased odds of developing complications (β = −0.793, odds ratio [OR] 0.452, 95% confidence interval [CI]: 0.25, 0.75, *p* = .004), when preoperative BOAS grade and gender were controlled.

**TABLE 4:** Multivariate logistic regression model of factors associated with major postoperative complications.

Variable	B (standard error)	Wald test statistic	*p*‐value	Estimated OR	95% CI for OR
Intercept	1.846	1.673	.094	21.922	0.456–904.249
Gender	0.671	−1.446	.148	0.379	0.094–1.381
BCS	0.275	−2.881	.004	0.453	0.251–0.749
Preoperative BOAS grade 1	1.352	−1.548	.122	0.123	0.010–3.084
Preoperative BOAS grade 2	1.272	−0.440	.660	0.561	0.056–13.170

Abbreviations: BCS, body condition score; BOAS, brachycephalic obstructive airway syndrome; CI, confidence interval; OR, odds ratio.

### 3.2 Respiratory functional grading and BOAS index pre‐ and postoperatively

**FIGURE 3:**
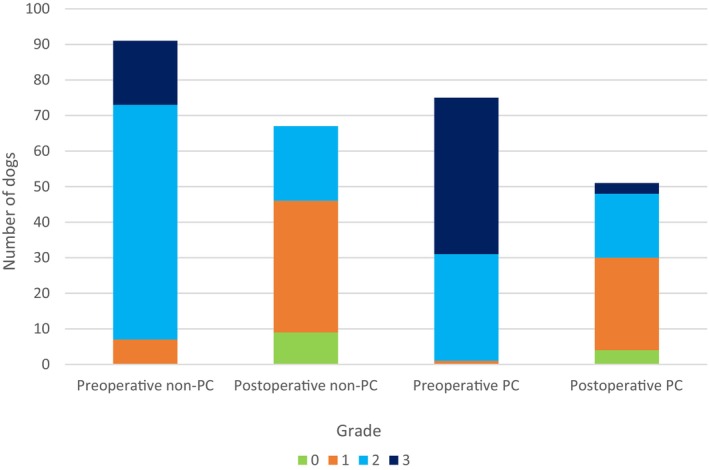
Respiratory functional grading scores.

**FIGURE 4:**
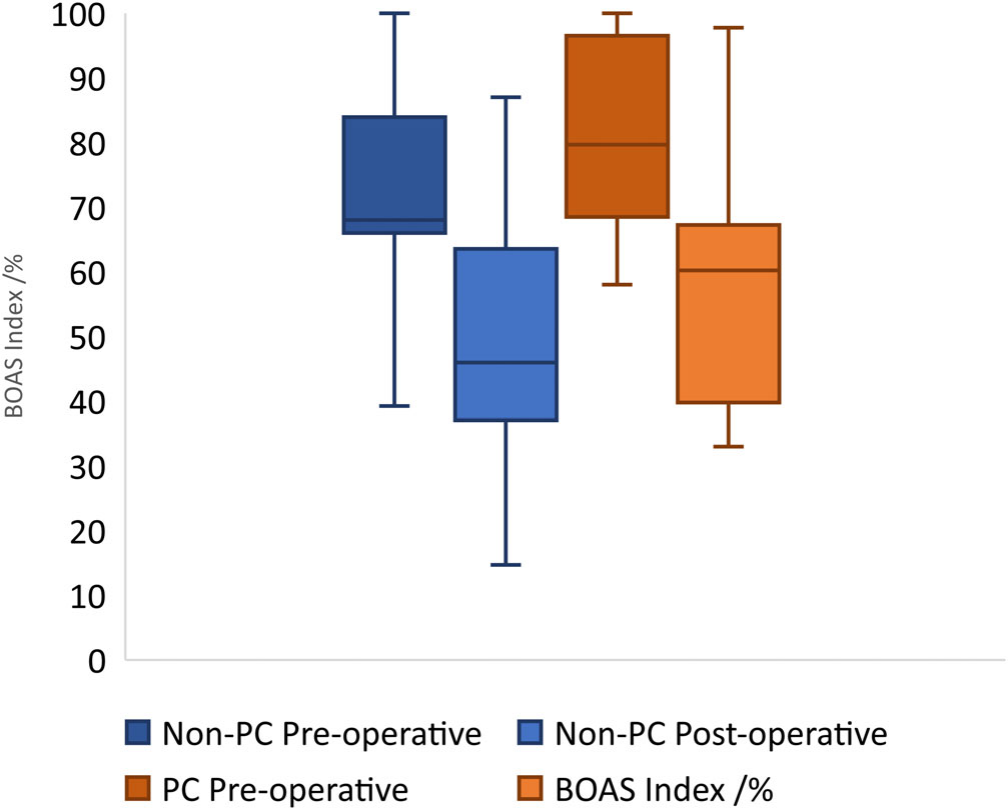
Pre‐ and postoperative brachycephalic obstructive airway syndrome index.

Preoperative respiratory functional grading results (Figure 3) were available for 166 study dogs, with postoperative results available for 118 dogs (51 PC; 67 non‐PC). The median preoperative respiratory functional grade was higher in the PC group (median = 3) than in the non‐PC group (median = 2, *p* < .0001), with both falling to one postoperatively and losing between group difference (*p* = .144). Functional grade decreased postoperatively overall (median reduction = 1 grade, V = 4095, *p* < .0001) and within analysis limited to the PC group (median reduction = 1 grade, V = 861, *p* < .0001). Pre‐ and postoperative BOAS indices were available for 95 dogs (30 PC; 65 non‐PC). BOAS index decreased significantly postoperatively in both the non‐PC group (median reduction = 20.7%, *p* < .0001) and the PC group (median reduction = 28.5%, *p* < .0001) (Table 1) (Figure 4).

## 4 DISCUSSION

The most notable finding in this study was that cuneiformectomy was not associated with a higher risk of either overall or major complications, particularly given that the population of dogs undergoing cuneiformectomy were more severely affected than the non‐PC group. The incidence risk of mortality of 3.5% in the PC group compared favorably with the 15% mortality recorded in previous studies.^31^ This may be explained first by the relatively atraumatic technique associated with the use of microvascular scissors and by limiting resection to the cuneiform process, thereby reducing the risk of obstructive laryngeal webbing and granuloma formation^27^ and second due to the reduction in inspiratory effort in the recovery period which in turn may lead to reduced airway inflammation. Some studies have found that aspects of laryngeal collapse, notably ventricle eversion, may recur over time^13^; it is unclear whether this could be the case with the resected cuneiform processes over time.

As previously discussed, there is a positive correlation between airway disease and gastrointestinal signs in brachycephalic dogs.^3^ The negative intrathoracic pressures occurring in these dogs during inspiration is argued to exacerbate gastroesophageal reflux; equally gastrointestinal signs such as regurgitation, vomiting and reflux can in turn induce persistent pharyngeal inflammation.^3^ The presence of gastrointestinal disease is often associated with weight loss; for instance, Liu et al.^19^ found that 15% of French Bulldogs with grade 3 laryngeal collapse were underweight and all of these had frequent regurgitation. Consistent with this, a lower BCS was found to be associated with increased risk of developing major complications in this study. Many studies classify dogs as being either overweight or not overweight. The latter group comprises normal and underweight dogs, consequently it is difficult to extricate from previous studies whether underweight dogs (BCS <4) specifically are associated with more severe disease and complications. A further consideration is that the risk of being clinically affected by BOAS is higher in overweight dogs, therefore underweight dogs comprise a minority of dogs with BOAS as found in this study. In this study, it was found that the incidence risk of complications for both overall and major complications were highest in underweight dogs and lowest in overweight dogs. It is conceivable that the underweight dogs were those with gastrointestinal disease and more susceptible to complications arising from this. However, given the small numbers of these dogs in the sample it is difficult to ascertain the exact relationship between these signs. Additionally, these signs may not have been identified in all cases and, given the retrospective nature of the study, may have been underreported.

Though it was not possible to extricate the additional benefit of performing cuneiformectomy over the other aspects of multilevel surgery, the significant improvement observed in grade in these dogs is promising and provides a valuable foundation for further studies to explore the benefit in more detail. As demonstrated in Figures 1 and 2, the opening of the glottis achieved with cuneiformectomy may reduce the resistance to airflow in dogs with higher grade laryngeal collapse. Though a reduction in BOAS index was noted in both groups, the lack of postoperative plethysmography recordings and the heterogeneity of the initial indices prevented further statistical analysis being performed to ascertain the additional benefit of cuneiformectomy over MMS in these dogs. The finding that the duration of hospitalization was no greater in the cuneiformectomy dogs is important to note given the morbidity previously associated with laryngeal surgery in other studies.

The study had several limitations. The non‐PC and PC groups comprised heterogenous populations as the latter group involved dogs with considerably more severe airway disease. The decision to proceed with cuneiformectomy was dependent on a clinical assessment of whether the case would benefit from the procedure. Data were lacking for RFG and BOAS index for some cases; in particular for the latter where some dogs would not tolerate plethysmography sufficiently to obtain repeated readings. Though major complications were noted, given the retrospective nature of the study, some clinical records may have been incomplete and some complications underreported. For instance, some of the complications data included subjective reports from owners and staff, for example concerning the degree of postoperative regurgitation. Yet there is no reason to believe that this limitation is more likely to apply either the non‐PC or PC dogs and consequently should not influence the overall finding that cuneiformectomy is not associated with a greater risk of complications. The lack of statistical difference found in complications between groups may be attributable to the study being underpowered rather than truly representing equivalence. Certain variables were not controlled for such as the surgeons performing the procedure or more minor variations in multilevel surgery such as the form of palatoplasty. However, the variability in surgeons involved in the study could help to improve the validity of the findings, negating the risk that the outcomes were heavily influenced by one individual surgeon's ability to perform the procedure.

Given that the cross‐sectional area of the rima glottidis is markedly reduced in BOAS dogs with laryngeal collapse, performing other forms of airway surgery is likely to be less effective unless this area is addressed. Though alternatives are available for surgical management of laryngeal collapse in BOAS dogs, the relative ease and simplicity of the procedure, with the advantage of preserving the normal function of the larynx, and without the requirement for repositioning of the patient, confer considerable advantages over alternatives such as arytenoid lateralization or permanent tracheostomy. The results that cuneiformectomy is not associated with a higher incidence risk of complications, even in more severely afflicted dogs, is an important finding. It is hoped that the results of this study and increasing awareness of this procedure will facilitate the management of higher‐grade laryngeal collapse in multilevel airway surgery.

## AUTHOR CONTRIBUTIONS

Chan AJ, BVSc (Hons), BA(Hons), MRCVS: Responsible for data acquisition, interpretation and analysis, and drafting and revision of the manuscript. Liu NC, DVM, MPhil, PhD: Responsible for data acquisition, statistical analysis and critically revising the work. Ladow JF, VetMB, CertVR, CertSAS, DipECVS, MRCVS: Responsible for study design, data acquisition and analysis and critically revising the work. All authors gave final approval for this version to be published and agree to be accountable for any aspect of the work. The authors would like to thank the owners and referring veterinarians of the dogs participating in this study in addition to our colleagues in the Queen's Veterinary School Hospital, University of Cambridge, particularly Laura Owen DipECVS and Julia Riggs DipECVS.

## CONFLICT OF INTEREST STATEMENT

The authors have no conflicts of interest to declare.
